# Imaging in translational cancer research

**DOI:** 10.20892/j.issn.2095-3941.2022.0677

**Published:** 2022-12-05

**Authors:** Felix T. Kurz, Heinz-Peter Schlemmer

**Affiliations:** ^1^Department of Radiology, German Cancer Research Center, Heidelberg 69120, Germany

**Keywords:** Cancer imaging, computed tomography (CT), magnetic resonance imaging (MRI), positron emission tomography (PET), cross-sectional imaging

## Abstract

This review is aimed at presenting some of the recent developments in translational cancer imaging research, with a focus on novel, recently established, or soon to be established cross-sectional imaging techniques for computed tomography (CT), magnetic resonance imaging (MRI), and positron-emission tomography (PET) imaging, including computational investigations based on machine-learning techniques.

## Introduction

For preclinical and clinical research and subsequent translation to routine applications in patient care, such as the development of novel targeted therapies for metastatic disease, diagnostic imaging has a pivotal role in disease detection, grading, treatment planning, therapy monitoring, and detection of recurrence^1^. With the availability of ever more powerful computational methods, new imaging biomarkers may emerge and shape future clinical decision-making tools, and may also be used in fundamental and translational research^2^. The current arsenal of tomographic imaging techniques is continually being updated and improved upon, thus leading to the development and establishment of new computed tomography (CT) techniques with improved contrast, and spatial and temporal resolution, as well as diminished radiation exposure^3^; novel imaging sequences in magnetic resonance imaging (MRI) for assessing, e.g., the specific microstructural composition of biological tissue *via* its diffusive properties^4^; new molecular markers to advance positron-emission tomography (PET) imaging^5,6^; improved hybrid imaging techniques combining PET and CT or MRI^7,8^; and novel imaging-sifting computational methods based on machine-learning and high-throughput analysis of large patient cohorts^9,10^.

Although many techniques may offer promising applications, their potential to be translated to routine clinical imaging is continually being evaluated or reconsidered, and translation is not ensured; some techniques, such as MRI spectroscopy, have been well-known for several decades but remain rarely used in routine imaging^11^. However, the role of MRI spectroscopy in oncological research is indispensable and may increase, on the basis of sequences with improved artifact reduction, resolution, and time efficacy^12^.

Typically, the interpretation of *in vivo* tomographic imaging in preclinical settings is compared with results from *ex vivo* histology and/or microscopy images with much better spatial resolution, as well as with patient characteristics, to obtain integrative correlative measures that may indicate disease status. On the basis of these results, the interpretation is extended to clinical imaging and serves as a basis for clinical decision-making. Animal studies may even be used to enable correlative approaches that compare *in vivo* tomographic imaging with *in vivo* microscopic imaging from, e.g., multiphoton microscopy^13^. These experimental imaging procedures are becoming increasingly important because they allow for the application and direct comparison of common clinical imaging techniques with ground truth microscopic images in a non-destructive *in vivo* setting, thus providing the benefit of a live view of functional disease properties otherwise not possible in human imaging.

## CT imaging

CT imaging is currently the most commonly used cross-sectional imaging method in radiological imaging, and is applied in emergency situations, screening protocols, and cancer staging examinations. In fact, most oncological staging procedures, particularly in thoracic and abdominal radiology, rely on standardized CT imaging protocols according to national consensus guidelines such as those from the association of the scientific medical societies in Germany (AWMF). CT imaging is rapid and allows for quantitative evaluation of neoplastic disease; however, it has the drawbacks of radiation exposure through X-rays and contraindication for patients with allergies to CT contrast agents and/or who are at risk of iodinated contrast-induced thyrotoxicosis or impaired renal function.

Beyond improving imaging resolution and speed, novel techniques in CT imaging have been aimed at decreasing radiation exposure and the need for contrast agent. Nonetheless, currently available commercial CTs are already approaching their physical limits in terms of technological advances in gantry, tube, and detector design^14^. The number of detector rows in the z-direction (along the patient long axis) is typically between 192 and 320; this number, together with the maximum detector panel width of 16 cm, is not expected to substantially increase in the coming years, because it already allows for adequate cardiac imaging within one heartbeat^15^. One recently established technique is CT-based measurement of the fractional flow reserve to model flow in coronary arteries by using anatomic physiological modeling, that can be used to quantify the extent of coronary artery disease, which is a potential radiation-therapy related side effect^16,17^.

Advances in tube technology saw the introduction of specific prefilters that selectively remove photons with low energy from the photon spectrum of the CT cone beam, thus decreasing radiation exposure and enabling faster scanning^18,19^. These advances allow for high-speed, low-kilovolt (kV) examinations that can accommodate patients with high body-mass index, and 100 kV has replaced 120 kV as the new imaging standard^20–22^. The decrease in radiation exposure is highly sought after for future screening exams. To date, low-dose CT screening is advised only for lung cancer screening in adults 50–80 years of age with a history of smoking of at least 20 pack years^23^, and remains under investigation for the detection of early lung cancer^24^.

### Photon counting detector CT imaging

**Figure 1:**
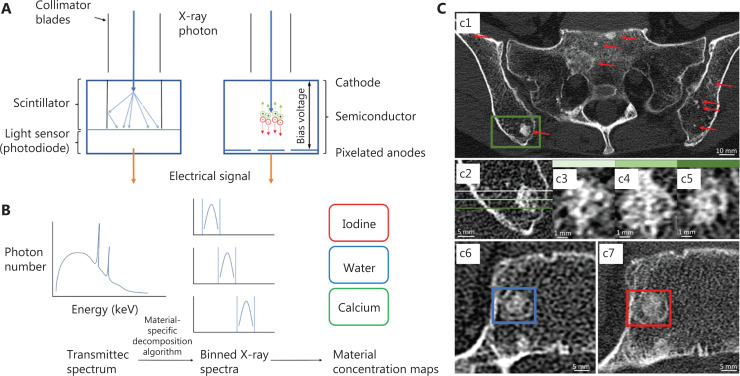
(A) Schematic representation of the integrated energy detector (EID; left panel) and the photon-counting detector (PCD; right panel). In the EID, X-rays enter a solid-state scintillator detector and convert to scintillation light through interaction. The resulting photons hit a photodiode, which converts the light to an electrical signal proportional to the sum of all X-ray photon energies. The PCD allows X-ray photons to interact with a semiconductor material layer, thus producing positive and negative charges. The negative charges are drawn to pixelated anodes and record individual X-ray photons directly in an electrical signal proportional to the photons’ individual energy. (B) The PCD allows for recording of different energy spectra by using energy thresholds (middle panel) that produce material-specific maps in a manner dependent on the material concentration within the tissue (for instance, iodine, water, calcium), on the basis of specific material decomposition algorithms. (C) Unenhanced EID (c6) and PCD (c1–c5, c7) images of a 68-year-old patient with unifocal medium-grade breast cancer and histologically confirmed osseous metastases (CT window: C = 500 HU, W = 1,500 HU). (c1) Red arrows indicate multiple osteoblastic metastases with one metastasis in the right iliac bone with a complex sclerotic composition pattern (c2–c5). (c6, c7) EID and PCD CT image of a metastasis in the right fifth lumbar vertebra. The gain in resolution and therefore diagnostic accuracy is clearly seen. Figures in (A, B) are adapted from Ref.^26^ (CC BY 4.0 license), and the images in (C) are adapted from Figure 3 in Ref. ^37^ (CC BY 4.0 license).

Developments in CT detectors have included the introduction of smaller detector elements as well as so-called photon counting detectors (**Figure 1**). In a conventional, energy-integrating detector, X-rays enter a solid-state scintillator detector, thus generating scintillation light through interaction, which hits a photodiode that converts the light into an electrical signal proportional to the sum of all detected X-ray photon energies, regardless of the individual photon energies^25^. In photon counting detectors (PCDs), however, the interaction of X-ray photons with the single layer semiconductor detector material (typically cadmium zinc telluride) produces positive and negative charges, wherein the negative charges are drawn to pixelated anodes and record individual X-ray photons, thus enabling direct conversion to an electronic signal proportional to the photons’ individual energies^26^. Because the signal is recorded within only nanoseconds, detector pixel sizes can be designed to enable photon energy discrimination in clinical CT imaging^27^. Measuring individual photon energies has high potential to be adapted by using elements such as bismuth, gadolinium, and iodine to produce new imaging contrasts and potentially enable multi-phase imaging within a single recording^28,29^.

The following contrasts are currently being investigated. PCDs in micro-CT imaging have been used in nanoparticle contrast agent imaging of mouse sarcoma with iodine and gadolinium nanoparticles, thus achieving improved visualization of the tumor vasculature and intratumoral distribution patterns of contrast agent nanoparticles^30^. In a similar study, iodine and gadolinium based intravenous contrast agent injections in rats have been demonstrated to feasibly differentiate peritoneal metastases in colorectal carcinoma with a specificity of 100%^31^. Furthermore, in a mouse model of soft tissue sarcoma receiving radiation therapy, the radiomic features from PCD CT with iodine contrast have been found to be superior to those of conventional micro-CT in differentiating among various tumor metrics^32^. Such functional characterizations of tumor tissue are expected to be important in future preclinical research and may be used in whole-body PCD CT imaging for translational research from rodents to humans^33^.

Owing to the smaller pixel sizes, the in-plane resolution and longitudinal spatial resolution are significantly better in PCD CT scanners than conventional CT scanners, and in-plane resolutions as high as 50–150 μm have been achieved^34,35^. This improvement enables better detection of small structures in biological tissue such as small bronchi^36^. Moreover, ultra-high-resolution PCD CT imaging has shown potential in detecting small osteolytic bone metastases in breast cancer that would have otherwise been missed by conventional imaging^37^. In addition, the improved resolution also allows for the detection of microcalcifications in breast cancer specimens with sensitivity and specificity comparable to those of digital breast tomosynthesis, with a voxel size of (50 μm)^3,35^.

The feasibility of decreasing radiation exposure in PCD imaging technology has also been demonstrated in lung tissue and shown higher contrast, lower noise levels, and ultimately better diagnostic quality than energy-integrating detector CT imaging^38^. Therefore, PCD CT imaging is expected to be particularly interesting in future low-dose lung cancer screening studies. A recent study has reported the development of a prototype PCD CT scanner with a relevant dental filling artifact reduction^39^; this method, if validated in clinical studies, would have enormous potential in head and neck cancer staging, wherein artifacts are the primary reason for cancer misclassification and recurrence of missed tumors^40^.

### Phase-contrast-based synchrotron CT imaging

**Figure 2:**
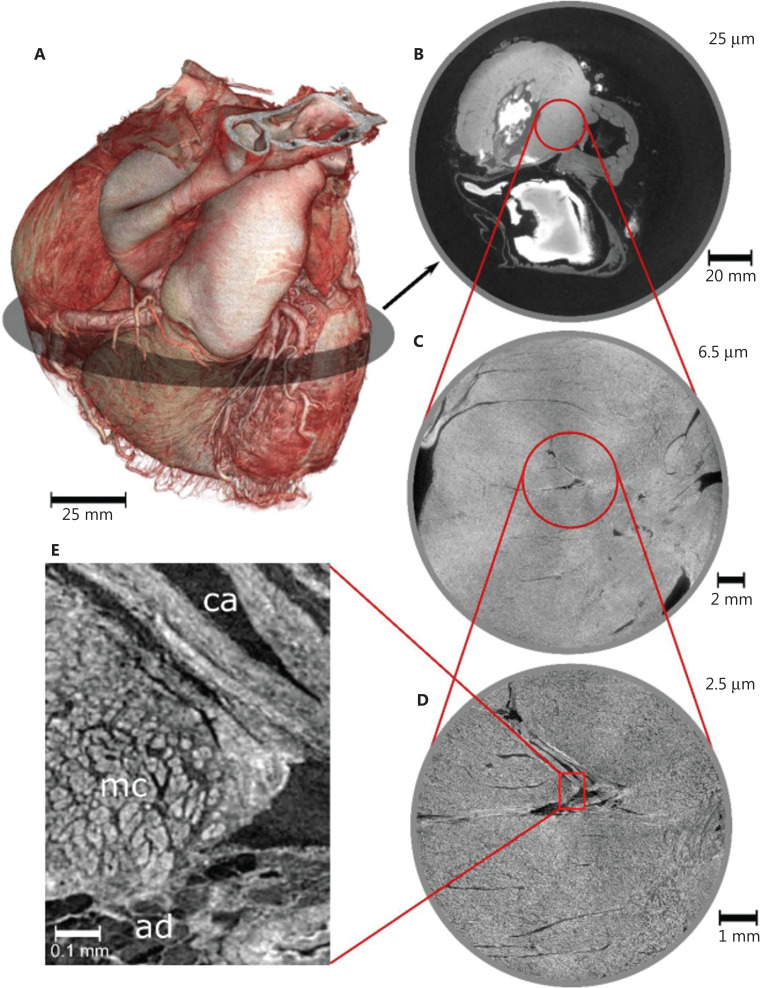
Phase-contrast-based synchrotron CT images of an intact human heart. (A) Intact human heart, and magnified cross-sections with voxel sizes of (B) 25 μm, (C) 6.5 μm, and (D) 2.5 μm, and (E) magnification of the image in (D), revealing the local arrangement of ventricular myocytes (mc), a coronary artery (ca), and adipose tissue (ad). Adapted from Ref.^233^ (CC BY-NC 4.0 license).

Another emerging technology in preclinical medical research is phase-contrast-based synchrotron CT imaging, performed at synchrotron radiation beamlines^41,42^. This method is sensitive to light elements (hydrogen, carbon, nitrogen, and oxygen) whose X-ray phase shift cross section is much larger than the X-ray absorption cross section; therefore, phase maps from an X-ray interferometer may be used to detect specific interference patterns and subsequently reveal microscopic soft tissue details at nanometer scale without a need for specific tissue staining^41^. Although such analyses have been restricted to only very small sections of biological specimens, the first high-energy (6-GeV), fourth-generation synchrotron source at the European Synchrotron Radiation Facility has allowed for the scanning of human organs in toto^42,43^ (**Figure 2**). This non-destructive technique may provide unprecedented detail of the extent of oncological disease, thus aiding in studies of tumor infiltration patterns and changes in the tissue microenvironment around tumor cells in whole human organs and whole rodents.

### Outlook

The exciting new possibilities of PCD CT imaging with increased resolution and decreased radiation exposure, as well as additional contrasts, are beginning to be explored in the first large-scale clinical studies, which are expected to shape the future of CT imaging^44,45^. Although only few PCD CTs are currently available, many more are expected to be found in hospitals and radiology cabinets. Their potential and pitfalls are expected to determine future oncological disease diagnosis, staging, and monitoring. Simultaneously, progress in *ex vivo* phase-contrast-based synchrotron CT imaging with imaging of whole human organs is expected to facilitate exploration of cancer growth patterns in greater detail, thereby providing a microstructural basis to detect, interpret, and quantify CT imaging patterns in oncology.

## MRI

**Figure 3:**
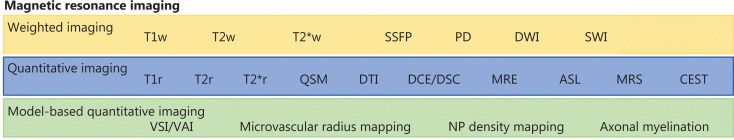
MRI sequences used in translational cancer research, including weighted sequences, quantitative sequences, and model-based quantitative microstructural imaging sequences. T1w, T1-weighted imaging; T2w, T2-weighted imaging; T2*w, T2*-weighted imaging; SSFP, steady state free precession imaging; PD, proton spin density imaging; DWI, diffusion-weighted imaging; SWI, susceptibility-weighted imaging; T1r, T1 relaxation mapping; T2r, T2 relaxation mapping; T2*r, T2* relaxation mapping; QSM, quantitative susceptibility mapping; DTI, diffusion tensor imaging; DCE, dynamic contrast-enhanced MR perfusion imaging; DSC, dynamic susceptibility contrast MR perfusion imaging; MRE, magnetic resonance elastography; ASL, arterial spin labeling imaging; MRS, magnetic resonance spectroscopy; CEST, chemical exchange saturation transfer imaging; VSI, vessel size imaging; VAI, vascular architecture imaging; NP, nanoparticles. Further details are in the main text.

MRI has markedly advanced since the first recorded nuclear magnetic resonance (NMR) signal in an anesthetized rat was reported by Jackson and Langham in 1968^46^. Raymond Damadian proposed and patented an “Apparatus and method for detecting cancer in tissue” relying on NMR technology in 1974, on the basis of his findings that the spin-lattice (T1) and spin-spin (T2) magnetic relaxation times differed between normal rat tissue and sarcoma and hepatoma tissue^47^. Likewise, other groups found differing NMR relaxation times between healthy and neoplastic tissue^48^. Several years after Paul Lauterbur produced the first MR images in 1973^49^, the first clinical MRI was introduced, thus eventually leading to the high-field machines with 1.5 Tesla and 3 Tesla magnetic field strengths in clinical use today. Simultaneously, apart from the fundamental T1- and T2-weighted sequences, many new MR sequences have been developed to elucidate various tissue properties. To comprehend their roles in translational cancer research, we distinguish between weighted and quantitative imaging MRI sequences (**Figure 3**).

In weighted sequences, the contrast can be optimized by varying specific sequence-inherent parameters, e.g., the echo time (TE) for T2-weighted sequences in the range of T2^50^. The typical weighted sequences are T1-, T2-, and proton density (PD) weighted sequences, with short repetition time (TR) and short TE, long TR and long TE, and long TR and short TE, respectively. Other weighted sequences are diffusion-weighted imaging (DWI) and T2*-weighted and/or susceptibility-weighted imaging (SWI) sequences, which were developed in the late 1980s and 1990s and are now part of the standard MRI protocols in cancer imaging. Steady-state free precession MRI sequences maintain a residual transverse magnetization between successive MRI pulse cycles to generate a mixed contrast between T2 and T2* weighting, and are used primarily in dynamically changing organs such as the heart^51^.

Quantitative sequences enable acquisition of tissue-specific physical entities or parameters that therefore are prone to changes with alterations in tissue composition, e.g., owing to metabolic changes or tumor cell infiltration. These sequences have a functional quality that cannot be attributed to conventionally structural sequences, with the sole exception of the apparent diffusion coefficient (ADC), a quantitative parameter obtained in DWI sequences. Apart from ADC mapping and dynamic-contrast enhanced (DCE) or dynamic susceptibility contrast (DSC) MR perfusion sequences, all these functional, quantitative sequences are not yet part of standard oncological MRI protocols. Their roles in translational cancer imaging are reviewed below.

In addition, model-based quantitative imaging methods provide indirect information on the voxel-inherent microstructure. They are motivated by small magnetic field inhomogeneities that produce local changes in magnetic susceptibility and diffusion, both of which influence the MR signal decay. Measurements of detailed MR signal decay curves therefore allow for quantification of the underlying microstructure, e.g., microvascular density or axonal myelination.

### DWI, diffusion-tensor imaging, and intravoxel-incoherent motion imaging

DWI is currently integral part of most MRI protocols in oncologic imaging for a variety of tumors, e.g., brain tumors, prostate cancer, or metastatic disease in general^52^. Increased tumor cellularity restricts the diffusion of water molecules around tumor cells, thus resulting in a hyperintense signal in DWI images with higher b-values. This sensitivity to diffusion results from the applied b-values whose quality depends on the gradient properties during the pulse sequence design. The role of DWI in differentiating malignant neoplastic tissue from healthy tissue or predicting treatment response has been investigated in most, if not all, tumor types^52–57^. Although its resolution (2–5 mm^2^ in plane) is typically lower than those of structural sequences, its b-value dependent contrast with cell-rich tumor tissue and its ability to provide tissue-specific ADCs have demonstrated great success in identifying tumor tissue. An increasing number of MRI tumor protocols now incorporate DWI in their tumor grading systems, most prominently the Prostate Imaging Reporting and Data System (PI-RADS) classification for prostate cancer^58^, the Breast Imaging Reporting and Data System (MRI BI-RADS) classification for breast cancer^59^, the Myeloma Response Assessment and Diagnosis system (MY-RADS) classification for myeloma^60^, and the Ovarian-Adnexal Reporting Data System (O-RADS) MRI score for ovarian cancer^61^.

Owing to its relatively fast acquisition time and superior contrast, DWI is also being investigated for its use in MRI cancer screening protocols, specifically in whole-body MRI cancer screening in cancer predisposition syndromes^62^. However, the roles of DWI and of MRI in general in screening common cancer types such as breast, prostate, and lung cancer remains controversial^63–65^.

Diffusion tensor imaging, in contrast to DWI, considers diffusion along multiple directions in three-dimensional space. Because water diffusion in biological tissue is often anisotropic, and not isotropic, i.e., equal in all directions, the concept of a diffusion tensor captures and quantifies this (fractional) anisotropy, thus enabling investigation of preferred tracts of diffusion, e.g., along neuronal fibers in brain tissue or peripheral nerves^66,67^. Although used primarily in neuro-oncology^68^, this method has also been shown to be beneficial in determining the histological grade of oral carcinoma^69^; discriminating between small-cell lung cancer and non-small cell lung cancer brain metastases^70^; correlating with Gleason scores in prostate cancer^71^; or predicting parametrial infiltration in cervical cancer^72^.

Diffusion within an MR voxel that differs from (extracellular) water diffusion, such as blood circulation within the capillaries, is termed intravoxel incoherent motion (IVIM). IVIM leads to attenuation of the diffusion MR signal at low b-values, because the diffusion coefficient, or pseudodiffusion, associated with blood flow is larger than the diffusion coefficient of water, thus effectively leading to a slightly larger ADC value^73^. Pseudodiffusion and diffusion can be separated, thereby enabling determination of a flowing blood volume fraction (fIVIM: fractional IVIM) to obtain estimates of blood perfusion without the use of external contrast agents (see Section 2.2.4)^74^. Owing to the abnormal microvasculature in tumors, IVIM is increasingly being applied in oncological settings^75^ mostly in rectal, pelvic, and hepatic cancers, and fIVIM has been found to strongly correlate with microvessel density^75,76^. Its role in common cancer types, such as breast and prostate cancer, is currently being evaluated^77,78^.

### Susceptibility-weighted imaging and quantitative susceptibility mapping

SWI is based on the differences in local magnetic susceptibilities in biological tissue, which lead to phase shifts at sufficiently large echo times^79,80^. However, across different MR vendors, SWI typically denotes a high-resolution susceptibility-enhanced sequence that is not necessarily processed with phase information, thus resembling a T2*-weighted sequence. This method is used in standard imaging protocols in neuroradiology to detect microbleeds or thrombi. However, it has rarely been used outside of neuroradiological imaging applications. Intratumoral susceptibility signals in brain tumors have been attributed to microbleeds and abnormal vasculature, and may aid in differentiating high- and low-grade gliomas^81,82^. This method has been shown to provide limited diagnostic benefit over T1-weighted sequences in the evaluation of melanoma brain metastases^83^, in which SWI artifacts inside metastases are attributed primarily to microbleeds. Recently, SWI has been used to differentiate osteolytic from osteoblastic spine metastases^84^; to show intratumoral hemorrhages in uterine sarcomas as a diagnostic differentiator^85^; or to evaluate treatment response in patients with prostate cancer receiving androgen deprivation therapy^86^.

Quantitative susceptibility mapping, the quantitative extension of SWI, has been proposed to provide local magnetic susceptibility values and evaluate their spatial distribution^87,88^. This method has been investigated as a potential biomarker of tumor severity in glioma, in which basal ganglia iron content increases with glioma severity^89^. Further studies are needed to assess its utility in oncological research and clinical imaging.

### T1-, T2-, and T2* mapping sequences

Relaxation time mapping sequences sample the MR signal decay to obtain measures for the relaxation times^90–92^. Although T1, T2, and T2* mapping techniques were initially used primarily in cardiac imaging to adequately characterize myocardial edema and disease^91^, or cardiac iron overload^92^, they have also been applied in oncology^93^. Their use beyond cardiac imaging has increased, owing to better reconstruction and k-space sampling techniques, e.g., for mapping peritumoral infiltration zones in glioblastoma and anaplastic astrocytoma with T2 mapping^94^, or to identify histological types of lung cancer with T1 mapping^95^. T1 mapping has also been used to predict Gleason scores in prostate cancer^96^, whereas T2 mapping in prostate cancer has been used to differentiate between healthy and cancerous gland tissue^97^.

The quantitative property of relaxation time maps, *via* a combination of several relaxation times, or ideally with other quantitative parameters such as ADC values, makes them well-suited to characterize heterogeneous tumor tissue and also to future robust extraction of tumor-intrinsic imaging patterns, e.g., in radiomics and deep-learning analyses of pathological tissue, as described below. A multiparametric quantification may uncover differences between specific disease subgroups within multidimensional parameter spaces that would otherwise not be detected, and therefore aid in further integrative diagnostic endeavors. The potential role of multiparametric MR mapping in breast, prostate, and liver cancer has been reviewed^98–100^. Quantitative parameters (T1, T2) from synthetic breast MRI have been found to discriminate among breast cancer receptor statuses or proliferation rates^101^, whereas combined T1/T2 relaxation time mapping, ADC mapping, tumor size, and cancer subtype modeling have been shown to effectively predict pathological response in breast cancer after one neoadjuvant chemotherapy cycle^102^. In prostate cancer, T2 mapping has revealed significantly lower T2 values in cancerous regions^103^, and has shown high diagnostic accuracy in differentiating between chronic prostatitis and cancer^104^. In contrast, T1 mapping has been found to predict Gleason scores^96^. In liver cancer, multiparametric MRI (T1, T2 relaxation times) has been found to be useful in detecting sinusoidal obstruction syndrome after oxaliplatin-based chemotherapy^105^. T2 relaxation times have been shown to be more sensitive and accurate in identifying malignant liver lesions than ADC values^106^, whereas T1 mapping has enabled differentiation among tumorous liver regions according to extracellular matrix composition in a rabbit hepatic cancer model^107^, as well as accurate detection of liver metastases in a mouse model at 7 Tesla^108^.

### MR perfusion and permeability sequences

Many tumors thrive with microvascular proliferation, most prominently the aggressive brain tumor glioblastoma, which requires MR techniques to image and quantify intra- and peritumoral perfusion or the permeability of leaky tumor vessels. DSC imaging typically uses single-shot (or multi-shot) echo-planar imaging with repetition times of 1–2 seconds over a contrast agent bolus. The local tissue contrast agent concentration can then be inferred from the altered MR signal (during contrast agent bolus passage) in relation to its baseline signal^109^, thus allowing for the calculation of perfusion parameters such as mean transit time, local blood volume and flow, and time-to-peak. DSC imaging is currently used almost exclusively in brain imaging to reveal cerebral hemodynamics and show increased blood volume in cerebral tumor tissue^110^. This method is part of the standard MRI protocol in glioma and other malignant brain tumors^111^. However, it has also been used to differentiate benign from malignant soft tissue tumors^112^, or benign from malignant head and neck tumors or parotid tumors^113,114^. Its potential to describe tumor vasculature in other tumor entities still must be explored.

DCE MRI uses baseline T1 mapping and subsequent acquisition of T1-weighted images during contrast agent bolus passage. The MR signal change can then be associated with the local contrast agent concentration *via* pharmacokinetic models such as the extended Tofts model^115,116^, which assume an exchange in contrast agent particles between the blood vessel plasma and the extravascular space, by using the plasma-extravascular space transfer constant K-trans as a measure for permeability. DCE MRI is particularly established in imaging of breast and prostate cancer, for which DCE is part of the standard MRI protocols, and its interpretation is included in the BI-RADS and PI-RADS criteria. This method is also frequently used in imaging intracranial tumor imaging^117^, liver imaging^118^, and head and neck cancer imaging^119^.

### Blood-oxygen-level-dependent MRI

The differing magnetic properties of oxygenized and deoxygenized hemoglobin were first described by Pauling and Coryell in 1936^120^: oxyhemoglobin is diamagnetic, whereas deoxyhemoglobin is paramagnetic, thus signifying their different magnetic susceptibilities. This effect was first used by Ogawa to develop a blood-oxygen-level-dependent (BOLD) imaging contrast between arterial (oxygenated) and venous (deoxygenated) blood^121^. Because firing neurons require blood-delivered oxygen to generate energy, in the so-called hemodynamic response, BOLD contrast can be used to detect task-activated brain regions in functional MRI (fMRI), which is extensively, although controversially, used in psychiatric and psychological studies^122,123^. However, typically on the basis of multi-gradient-echo sequences, BOLD imaging is easily implementable in clinical routines, and it can be used to measure tumor hypoxia, a hallmark of cancer associated with therapy resistance and tumor progression^124^. Hypoxia also affects the T1 relaxation time in tumors, thus giving rise to tissue-oxygen-level-dependent (TOLD) MRI contrasts, wherein an inhaled gas challenge between air and 100% oxygen leads to heterogeneous changes in T1 relaxation rates within the tumor tissue^125^. For instance, a strong association the between BOLD response and partial pressure of oxygen in prostate cancer, and between TOLD and delayed tumor growth after irradiation therapy^126^, have been demonstrated. More recent developments include the combination of TOLD and DCE MRI to identify tumor regions refractory to oxygen challenge within renal cancer xenografts, to more accurately determine tumor hypoxia^127^; the differentiation between musculoskeletal tumors through power spectrum analyses of the BOLD time series signals^128^; and the induction of cerebrovascular dysregulation, according to glioma grade, through time-shifted fMRI traces^129^.

### Magnetic resonance elastography (MRE)

MRE is a phase-contrast-based MRI method that quantifies the mechanical properties of biological tissue, such as elasticity or stiffness. Because the stiffness changes in most tumors can be quantified by the calculation of a shear and elastic modulus, MRE is increasingly being used in experimental oncological imaging, e.g., in brain tumors^130^. It relies on the generation of mechanical waves, which are generated with dedicated vibrating devices placed on patients’ bodies^131^. MRE functions with many pulse sequences, including spin echo and gradient-recalled echo sequences, with or without echo-planar imaging, and is typically performed at frequencies between 20 and 100 Hz in clinical imaging, and 200 and 1,500 Hz in preclinical imaging, preferably with 3.0 Tesla scanners to achieve higher signal-to-noise and contrast-to-noise ratios^131^. The resolution of MRE is usually lower than that of conventional MR images, and it assumes an isotropic material composition, which is clearly not the case for many tissue types such as white matter fibers or muscle tissue. Like ultrasound elastography, MRE is used in breast imaging to detect early changes in tissue stiffness due to neoplastic disease^132^. However, most applications to date have been in brain and liver imaging^130,133^. Recent studies have indicated that MRE can be used to assess therapy outcomes and tissue damage before and after microwave ablation of liver tumors^134^, and liver stiffness has been found to be a predictor of early recurrence in hepatocellular carcinoma after therapy^135^. Simulation and phantom studies of MRE imaging have linked MRE parameters to intratumoral pressure^136^, and experimental preclinical studies have indicated that MRE may be suitable for specifically studying tumors with nondestructive growth patterns, such as prostate cancer^137^.

### Arterial spin labeling (ASL)

ASL technique does not require external contrast agent but instead uses labeling of blood water spins in larger arteries with specific radiofrequency pulses to obtain perfusion measures, such as blood flow and blood volume in downstream tissue areas, through the subtraction of labeled and control images^138^. Several ASL techniques have been proposed. The most common variant is pseudocontinuous ASL, which uses multiple short pulses during continuous labeling of water molecules that pass through a labeling plane. Most applications of ASL are in translational or preclinical research; however, some institutes use ASL measurements to assess perfusion in pediatric tumors without a need for contrast agent administration^139^. Owing to difficulties in finding adequate labeling planes, ASL is typically used in brain, head and neck, and renal tumors^140–143^, but has also been investigated in other organs, such as the liver^144^, heart^145^, and prostate^146^. The low resolution of ASL images with respect to that of conventional MR sequences limits its use in oncological screening; however, the benefit of avoiding contrast agent administration is expected to result in increased use of this technique in pediatric imaging, in oncological patients with many follow-up exams, and in oncological wards aiming to replace contrast-agent-based perfusion MRI with less expensive imaging. Novel pulse sequence designs and deep learning techniques to improve ASL robustness and accuracy are currently being developed^147^.

### Magnetic resonance spectroscopy (MRS) and chemical exchange saturation transfer imaging

MRS measurements enable probing of metabolic tissue properties *in vivo*. MRS is frequently used in clinical settings, e.g., to determine metabolite content in brain tumors^148^. This method is based on a chemical shift of the local resonance frequency in the external, static magnetic field, because of the shielding effect of electron clouds that are associated with bonding electrons of the prevalent molecules within the tissue. The effect is proportional to the magnetic field strength and therefore is particularly relevant at high and ultra-high field strengths, such as 3 Tesla and 7 Tesla. The resulting spectrum of molecule peaks reveals the molecular composition of the probed tissue. For instance, in aggressive brain tumor glioblastoma, increases are observed in the peaks of choline, a marker of cellular membrane turnover; lactate, a marker of anaerobic metabolism; and lipids, a markers of severe tissue damage and necrosis, whereas N-acetylaspartate (NAA), a marker of neuronal viability, is decreased^149^. The use of MRS is often limited to low-grade gliomas in neuroimaging, to refine lesion differential diagnostics, as well as to single large tissue voxels to produce robust and reproducible spectra; otherwise, MRS is used primarily in research studies. However, with advanced MRS processing techniques, whole-brain MRS is clinically feasible and may be used to study large-scale metabolic alterations during tumor growth^12^.

Furthermore, MRS has been applied in many other tumors, particularly prostate cancer, thus revealing low levels of citrate, a key metabolite in oxidative phosphorylation^150^, in colorectal cancer^151^, cancers of the head and neck^152^, or breast cancer with elevated choline peaks^153^.

The recent development of chemical saturation exchange (CEST) imaging in brain tumors, e.g., to identify genetic markers such as isocitrate dehydrogenase mutation status or 1p/19q deletion status in gliomas, has received substantial attention in the oncological imaging community^154^. CEST imaging enables mapping of tissue pH levels *via* amide proton transfer CEST^155^, wherein backbone amide protons of mobile proteins and peptides provide the endogenous CEST contrast^156^. Further applications include glucose CEST (glucoCEST)^157^, to detect intercellular glucose delivery and transport; the first applications outside brain tissue, e.g., detection of ductal pancreatic adenocarcinoma^158^; kidney assessment *via* renal pH levels^159^; prostate cancer detection^160^; and diagnosis of breast cancer *via* glucosamine CEST in clinical settings^161^.

### Advances in cardiac MRI

Cardiac imaging in oncology is used not only to characterize cardiac malignant masses, but also cardiac dysfunction or cardiotoxic effects associated with cancer therapy, including peripheral vascular disease and vascular dysfunction^162,163^. Recent advances in cardiac MRI have seen the development of simultaneous multi-parametric acquisition and reconstruction techniques (SMART) to accelerate acquisition times in cardiac imaging. SMART is particularly helpful in patients with complicated heart rhythms or with poor compliance during breath-hold instructions^164^. Simultaneous collective acquisition of several MR parameters such as T1 and T1 relaxation time would therefore significantly improve cardiac MRI efficiency^165^. Further new and emerging imaging techniques include electrocardiogram gated double inversion recovery fast spin echo sequences (or “black-blood” sequences), which can more reliably be used for characterization of cardiac masses or detection of cardiac edema due to nulling of blood^166^; detection of coronary artery disease, as a cardiotoxic effect of cancer-associated therapy with high speed coronary magnetic resonance angiography or ultrafast imaging of the coronary arteries^167,168^; visualization of pulmonary arteries and their implication in oncological disease through 3D pulmonary MR angiography^169^; and evaluation cardiac flow velocities at high spatial and temporal resolution with four-dimensional flow MRI^170^. These techniques are currently being established to, e.g., evaluate the role of anthracycline-induced cardiotoxicity^171^, or detect early cardiotoxicity during immuno- and radiotherapy^172^.

### Model-based quantitative imaging

**Figure 4:**
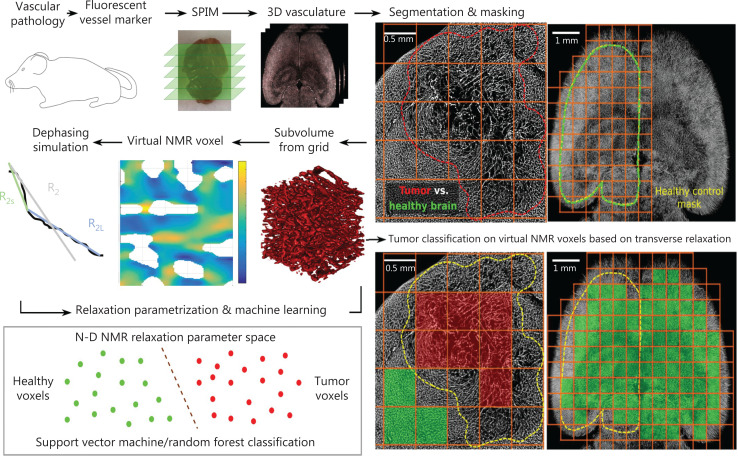
Virtual NMR voxel tumor classification. Left panel, top row: mice are injected with a fluorescent vessel marker, and subsequent brain resection, brain clearing, and selective plane illumination microscopy (SPIM) are used to visualize three-dimensional microvasculature; details have been described.^186^ Abnormal microvasculature in the tumor area (here, U87 glioblastoma) is delineated and partitioned into voxels with a 0.5 mm side length, which correspond to a voxel in a high-resolution MRI image stack. Similarly, a control mask is created on a healthy hemisphere and partitioned as well (far right, top row). Left panel, middle row: the microvasculature in each voxel is segmented, and spin dephasing is performed on the specific vascular arrangements to reveal multi-exponential MR signal decays (indicated with a MR signal decay relaxation rate component for short times, R_2s_, and for long times, R_2L_). Subsequently, relaxation parametrization occurs for each voxel, and can be used to train the classification of voxel vascular geometries into healthy and cancerous geometries (lower row) by using support vector machines or random forest classifiers; see also Ref.^185^. Adapted from Ref.^186^ (CC BY 4.0 license).

NMR signal decay is influenced by local magnetic susceptibility gradients and water diffusion, both of which depend on the microstructural arrangement of tissue, as first described 3 decades ago for local susceptibility differences between blood-filled idealized, i.e., cylindrical, vascular structures and static water molecules (static dephasing)^173^. This method is still widely used in MR signal decay simulations. However, MR signal modeling can now be used for increasingly complex arrangements of vessels and/or small particles such as iron-oxide nanoparticles^174,175^, and with the inclusion of diffusion effects^176–181^, in microstructural quantification *in vivo*^182,183^, also termed histological MRI (hMRI)^184,185^. Correlative analyses of MR signal decay and microvascular arrangements in tumor tissue can be studied with these modeling approaches and dedicated microscopy experiments (**Figure 4**)^185–187^. Similarly, myelinated axons and fiber tracts can be quantified by using the effects of anisotropic susceptibility within and around the myelin sheath^188,189^, and advanced models of microstructure derived from diffusion-weighted imaging, using multi-compartment environments as in nerve fibers, have been described^190–193^.

However, the increasingly complex theoretical models are often too computationally intensive to be implemented in routine clinical imaging. Moreover, multiparameter models may produce relevant fitting errors that do not allow for robust and reliable microstructure quantification. This gap is being filled by empirical signal models that obtain information on microvascular arrangements *via* surrogate parameters, such as vessel size and vessel architectural imaging (VSI, VAI)^194–196^. These methods are being used to detect microvascular changes, e.g., during antiangiogenic treatment in glioblastoma^197^.

## Hybrid imaging: positron-emission tomography CT and MRI

**Figure 5:**
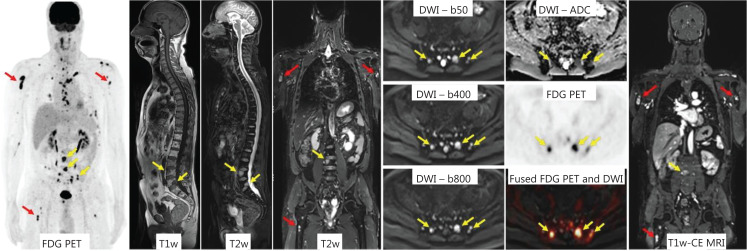
PET-MRI in a patient with multiple myeloma. Focal bone lesions are visible in the axial skeleton (yellow arrows) and in the proximal upper and lower extremities (red arrows) in ^18^F-fluorodeoxyglucose (FDG) PET, in DWI images at different b-values (b = 50, b = 400, b = 800), in the ADC map, and in fat saturated contrast-enhanced T1w images (T1w-CE MRI). The combined structural, metabolic, and MR functional (DWI) information enables multiparametric, multifunctional characterization of the multifocal disease activity. Adapted from Ref.^200^ (CC BY 4.0 license).

PET is a functional imaging method that uses radioactive tracers to visualize biological processes within the human body. The radiotracers frequently used for routine PET imaging in oncology are ^18^F-fluorodeoxyglucose (FDG) and prostate-specific membrane antigen (PSMA); their roles in cancer phenotyping and recommendations for their use in oncological imaging have been reviewed^198,199^. Increased local FDG uptake in the PET images reflects increased regional glucose transporter activity on a cellular level, which in turn indicates increased cellular metabolic activity (**Figure 5**)^200^, as observed as an unspecific finding in a variety of metabolic active and growing cancers and their metastases. Consequently, FDG-PET imaging is a valuable tool to provide diagnostic certainty when CT or MRI morphologic findings are otherwise unclear and lack functional information on biologic activity. In contrast, CT and MRI add essential anatomic information that PET is lacking. Hybrid imaging approaches are therefore synergistic: PET has been combined with CT (PET-CT) or MR (PET-MR) to enable fusion of CT and MRI images with areas of increased metabolic activity. PET-CT is the modality of choice for the staging and therapy monitoring of many cancers, such as head and neck, breast, lung, esophageal, colon cancers, melanomas, and aggressive lymphomas^201–203^. PET-CT imaging is currently being investigated for its clinical benefits in a wide range of malignant tumors, e.g., melanoma staging^204^, bone and soft tissue tumors^205,206^, and distant metastases and recurrence of head and neck tumors^206^.

PET in combination with MRI is available only at few hospitals and research centers. However, the prospects of correlating metabolic functional imaging with functional and structural MRI parameters may be attractive not only for answering research questions but ultimately for translation to clinical imaging^207^. E.g., FDG-PET-MRI has been found to outperform MRI in evaluating tumor size and nodal metastases in rectal cancer^208^, and MRI and CT for nodal staging in breast cancer^209^.

Apart from FDG and PSMA, several other tracers are currently being used and investigated in PET imaging, most prominently FAPI, which uses the tumor expression of fibroblast-activating protein (FAP) to diagnose and stage patients with cancers of the stomach or pancreas, and cholangiocarcinoma^210^. Other tracers are ^18^F-fluoroethyl-L-tyrosine (FET) and ^11^C-methionine (MET), which correlates with microvessel density in proliferating cells, both of which are used in glioma imaging; ^68^Ga-DOTATOC (DOTA^0^-Phe^1^-Tyr^3^-octreotide), a somatostatin analog used to detect neuroendocrine tumors^211^; and ^11^C-metomidate PET, which is used to detect adrenal masses^212^.

## Medical image computing in translational cancer research

**Figure 6:**
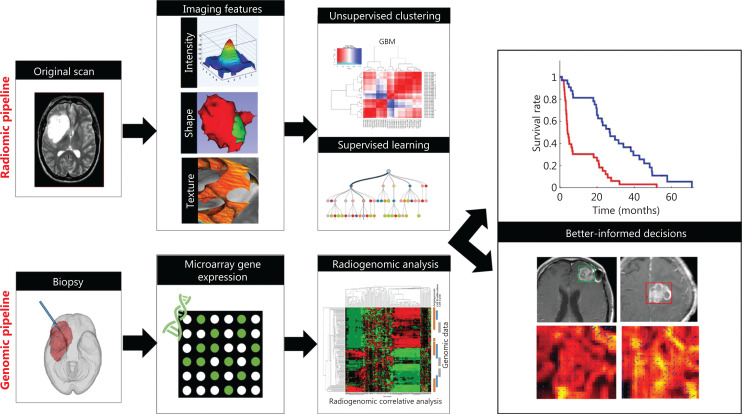
Standard radiomics pipeline for brain tumors. Top row: the original scan is normalized, and imaging features are extracted on the basis of IBSI criteria^230^. Subsequently, both unsupervised or supervised learning can be performed to classify radiomic features (RF) into those that correlate with therapy responders or non-responders, or with overall survival. Bottom row: gene expression profiles from tumor biopsies can likewise be screened for correlations with RFs to eventually identify RFs that may predict therapy success, recurrence, or overall survival, and therefore may affect clinical decision-making. Adapted from Ref.^222^ (CC BY 4.0 license).

Recent years have seen an astonishing increase in supervised and unsupervised computational medical image analyses to detect, segment, and classify tissue pathologies in oncology^213^. Together with other high-throughput analyses such as genomics, proteomics, or metabolomics, artificial intelligence based methods such as radiomics and deep-learning techniques have emerged as powerful tools in oncology. These methods enable extraction and quantification of imaging characteristics in radiological imaging, specifically pattern or texture analysis, which computationally allocates imaging signatures to pathological imaging changes^214,215^. Combined radiomics features (RF) can be used to predict disease status and changes during specific therapy regimens, on the basis of machine learning approaches in high-throughput agnostic analyses (**Figure 6**)^216^. Because RF based imaging is increasingly commonly used and, access to computational equipment and powerful computational hardware is increasing, RF based image interpretation is expected to be applied in clinical diagnostics in the coming years^217^.

Initially, RF extraction was performed in neuroimaging, e.g., to achieve radiomic profiling of glioblastoma tissue to identify imaging predictors of patient survival and anti-angiogenic treatment response^218,219^. Many other studies have applied RF extraction to all types of tumors and imaging modalities, most prominently lung tumors^220^, often in combination with genomic profiling, termed radiogenomics^221^. In many cases, relevant RFs have been found to discriminate between different tumor classes, e.g., low- and high-grade gliomas^222^; to be highly sensitive to cancerous tissue as in breast cancer^223^; or to predict treatment response in prostate cancer^224^. Several prospective clinical trials are currently underway to test the reliability and reproducibility of RFs in different centers and for machines of different vendors^225^. However, radiomics has not yet been applied in clinical imaging.

Developments in machine learning algorithms *via* artificial neuronal networks with many layers, or deep learning, often with so-called convolutional deep neural networks, have generated networks that can be trained to independently detect, segment, or classify image-based pathologies^226,227^. The recent explosion of deep learning studies in oncology has been due to increasing access to large databases of image material, such as the Cancer Imaging Archive of the NIH National Cancer Institute; to technological developments in graphics processing units; and to a vibrant and dynamic community spanning diverse disciplines such as mathematics, computational engineering, radiology, biology, or physics.

Although the applications of machine learning in medical image computing are manifold and promising, one major disadvantage is the lack of reproducible results, which may be attributed to the non-standardized RF parameters in radiomics, or to a non-negligible inter-scan variability with different acquisition parameters (e.g., in MRI, different flip angles, matrix size, TR/TE variations, field strength, etc.); non-expert annotation of imaging data and therefore annotation bias; and different and/or non-standardized machine learning models, many of which are prone to overfitting^228,229^.

For radiomics, the research community therefore has sought to establish standardized parameters in RF selection *via* the image biomarker standardization initiative (IBSI)^230^, and to build large databases to provide access to sufficiently large medical (anonymized) patient databases to allow broad testing of new algorithms. One commonly cited example is the Cancer Genome Atlas Glioblastoma multiforme data collection^231^.

Yet, with increasingly complex, often deep-learning-based, predictive modeling approaches, radiomics and deep learning feature selection is a purely statistical concept that focuses on predictive power rather than the biological meaning of these imaging characteristics, thus increasing the disconnect between radiological diagnostic decision-making in routine clinical practice and RF-informed image interpretation. This gap is expected to affect, and eventually limit extensive translation into routine clinical imaging^232^.

## Conclusion

The ongoing advances in cross-sectional imaging have significant impact on the progress in translational cancer research. New developments such as CT photon-counting detectors, multiparametric microstructural MRI, multimodal and hybrid imaging approaches, as well as their combination with machine-learning-based image analysis methods provide increasingly detailed insight into tumor biology and heterogeneity. Integrative assessment with the aid of bioinformatics enables combining structural imaging signatures with functional imaging characteristics, genomic or metabolomic profiles, and various patient clinical data. These advances in oncologic imaging are key pillars for future precision oncology.
